# Re-description of *Sinopodacurva* Zhong, Jäger, Chen & Liu, 2019 (Araneae, Sparassidae), with a first description of the female

**DOI:** 10.3897/BDJ.13.e152100

**Published:** 2025-03-25

**Authors:** Lijun Gong, Yang Zhong

**Affiliations:** 1 School of Nuclear Technology and Chemistry & Biology, Hubei University of Science and Technology, Xianning, China School of Nuclear Technology and Chemistry & Biology, Hubei University of Science and Technology Xianning China

**Keywords:** DNA barcode, huntsman spiders, morphology, taxonomy

## Abstract

**Background:**

*Sinopoda* Jäger, 1999 is a relatively large spider genus that currently comprises 141 species distributed worldwide. However, the genus remains inadequately studied because nearly half of the species are known from a single sex or juvenile specimens. *Sinopodacurva* Zhong, Jäger, Chen & Liu, 2019 was described, based on two male specimens from Damingshan National Nature Reserve, Guangxi Zhuang Autonomous Region, China and no additional specimens have been recorded since.

**New information:**

Recently, new materials of huntsman spiders have been collected from Mt. Wuyishan, including specimens of both sexes. Several males were identified as *S.curva*, based on morphological comparison with the holotype. Based on morphological characters and DNA barcodes, we confidently matched the females and males as *S.curva*. Herein, *S.curva* is re-described, based on these new materials and the female is described and illustrated for the first time.

## Introduction

*Sinopoda* Jäger, 1999 ranks as the fourth most species-rich genus within the huntsman spider family Sparassidae Bertkau, 1872, following *Pseudopoda* Jäger, 2000 (269 species), *Heteropoda* Latreille, 1804 (211 species) and *Olios* Walckenaer, 1837 (166 species) (World Spider Catalog 2025). With 141 described species to date, *Sinopoda* is primarily distributed across eastern Asia. Of these, 91 species are recorded from East Asia, 50 from Southeast Asia and one species from India (Zhang et al. 2023, Zhang et al. 2024). Notably, China is home to the largest number of *Sinopoda* species, with 75 species documented, accounting for 53.19% of the global total species (World Spider Catalog 2025). Most *Sinopoda* species have been well studied, particularly those newly described in recent years. These species have been described in detail, alongside high-quality illustrations, to allow easy species recognition (Zhang et al. 2015, Zhong et al. 2017, Zhong et al. 2018, Zhong et al. 2019, Grall and Jäger 2020, Zhu et al. 2020, Wang et al. 2021, Zhang et al. 2021, Chae et al. 2022, Zhong et al. 2022, Zhang et al. 2023, Zhang et al. 2023, Zhang et al. 2024). Despite this, alpha taxonomy of *Sinopoda* remains inadequately studied due to nearly half of the species having only been described, based on a single sex (13 from males only, 52 from females only) (World Spider Catalog 2025).

Recently, the authors examined sparassid specimens collected from Mt. Wuyi of Fujian Province, China and found several *Sinopoda* specimens containing both sexes collected from the same location, which are sharing similar habitus, markings, leg spination and other characters. It is very likely they are the opposite sexes of the same species. Based on comparison with the type specimen, we identified the male as *S.curva*. DNA barcodes (a partial fragment of the mitochondrial cytochrome oxidase subunit I gene, COI) of the new materials were also obtained to confirm gender matching. Based on both morphological and molecular data, we have matched the females and males as *S.curva*. The aim of the current paper is to re-describe the male and report the female for the first time, providing detailed morphological descriptions and illustrations.

## Materials and methods

Specimens in this study were collected by hand. Spiders were fixed and preserved in 95% ethanol. All the examined materials are deposited in the School of Nuclear Technology and Chemistry & Biology, Hubei University of Science and Technology (**HUST**), in Xianning, Hubei, China.

Specimens were examined using an Olympus SZX7 stereomicroscope; details were studied with an Olympus BX41 compound microscope. Male palps and female epigynes were examined and illustrated after being dissected. Epigynes were removed and cleared in warm lactic acid before illustration. Photos were taken with a Cannon EOS70D digital camera mounted on an Olympus BX43 compound microscope. The digital images were taken and assembled using Helifocus 3.10.3. software package (Khmelik et al. 2005).

All measurements were obtained using an Olympus SZX7 stereomicroscope and given in millimetres. Eye diameters were measured at the widest part. The total body length does not include the chelicerae or spinnerets. Leg lengths are given as total length (femur, patella, tibia, metatarsus, tarsus). Numbers of macrosetae are listed for each segment in the following order: prolateral, dorsal, retrolateral and ventral (in femora and patellae, ventral spines are absent and the fourth digit is omitted in the spination).

DNA barcodes were also obtained for matching. A partial fragment of the mitochondrial cytochrome oxidase subunit I (CO1) gene was amplified and sequenced for three specimens, using the primers C1-J-1718 (5’-GGAGGATTTGGAAATTGATTAGTTCC-3’) and C1-N-2776 (5’-GGATAATCAGAATATCGTCGAGG-3’). For additional information on extraction, amplification and sequencing procedures, see Zhang et al. (2021). All sequences were analysed using BLAST and are deposited in GenBank.

The distribution map was generated with ArcGIS ver. 10.5 (ESRI Inc 2002). The terminology used in the text and figure legends follows Zhong et al. 2019, Grall and Jäger 2020 and Zhang et al. 2023.

## Taxon treatments

### Sinopoda curva

Zhong, Jäger, Chen & Liu, 2019

9BEB49B0-6A1A-53C9-BE89-8BDCB33F39C5

*Sinopodacurva* Zhong, Jäger, Chen & Liu, in Zhong et al. 2019: 23, figs. 18A-C and 19A-F (description of male).

#### Materials

- **Type status:** Holotype. **Occurrence:** recordedBy: Yang Zhong; individualCount: 1; sex: 1 male; lifeStage: 1 adult; preparations: whole animal (ETOH); occurrenceID: E3D36967-560E-5B31-B99F-96F28F8C8046; **Taxon:** scientificName: *Sinopodacurva*; order: Araneae; family: Sparassidae; genus: Sinopoda; specificEpithet: *curva*; taxonRank: species; scientificNameAuthorship: Zhong, Jäger, Chen & Liu, 2019; taxonomicStatus: accepted; **Location:** continent: Asia; country: China; countryCode: CHN; stateProvince: Guangxi; county: Wuming; locality: Damingshan National Nature Reserve; verbatimElevation: 603 m; decimalLatitude: 23.53; decimalLongitude: 108.37; georeferenceProtocol: label; **Identification:** identifiedBy: Yang Zhong; dateIdentified: 12-12-2024; identificationReferences: Zhong et al. 2019; **Event:** samplingProtocol: hand picking; samplingEffort: 10 km by foot; eventDate: 27/5/2017; year: 2017; month: 5; day: 27; **Record Level:** language: en; basisOfRecord: PreservedSpecimen
- **Type status:** Other material. **Occurrence:** recordedBy: Yang Zhong; individualCount: 3; sex: 1 male, 2 female; lifeStage: 3 adults; preparations: whole animal (ETOH); associatedSequences: PV341275; PV341276; PV341277; occurrenceID: 7556B420-7D2E-5F04-BB22-F042B9240AB7; **Taxon:** scientificName: *Sinopodacurva*; order: Araneae; family: Sparassidae; genus: Sinopoda; specificEpithet: *curva*; taxonRank: species; scientificNameAuthorship: Zhong, Jäger, Chen & Liu, 2019; taxonomicStatus: accepted; **Location:** continent: Asia; country: China; countryCode: CHN; stateProvince: Fujian; municipality: Wuyishan; locality: Wuyishan National Nature Reserve; verbatimElevation: 1200 m; decimalLatitude: 27.58; decimalLongitude: 117.48; georeferenceProtocol: label; **Identification:** identifiedBy: Yang Zhong; dateIdentified: 12-12-2024; identificationReferences: Zhong et al. 2019; **Event:** samplingProtocol: hand picking; samplingEffort: 10 km by foot; eventDate: 23/12/2023; year: 2023; month: 12; day: 23; **Record Level:** language: en; basisOfRecord: PreservedSpecimen

#### Description

**Figure 1.**
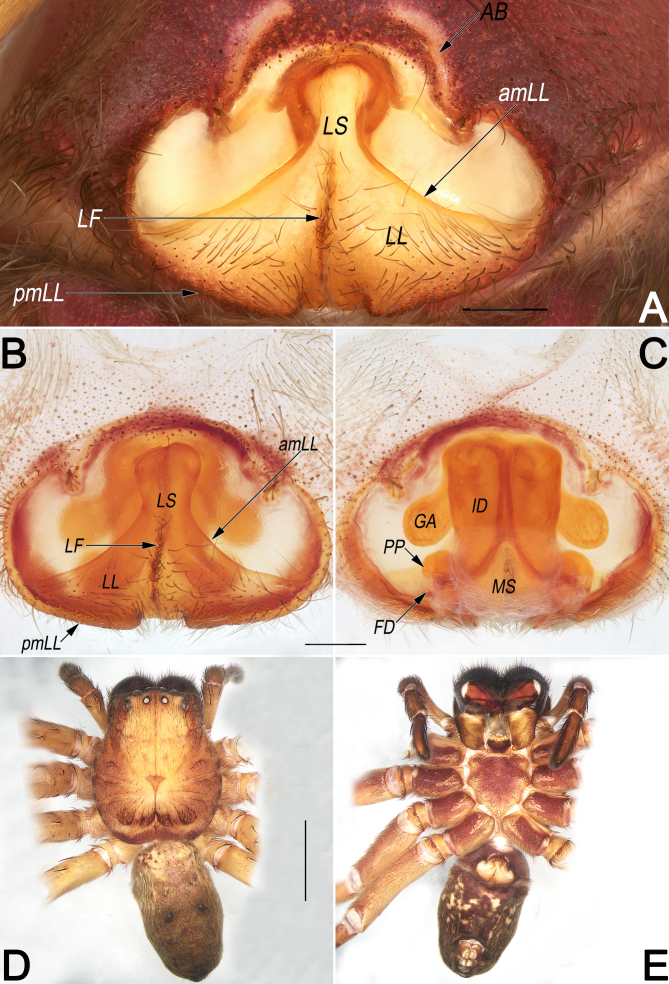
*Sinopodacurva* Zhong, Jäger, Chen & Liu, 2019 from Wuyishan National Nature Reserve. female, epigyne (**A–C**) and habitus (**D, E**). **A** intact, ventral; **B** cleared and macerated, ventral; **C** cleared and macerated, dorsal; **D** dorsal; **E** ventral. Abbreviations: AB = anterior band; amLL = anterior margin of lateral lobes; FD = fertilisation duct; GA = glandular appendage; ID = internal duct; LL = lateral lobe; LF = lobal fovea; LS = lobal septum; MS = membranous sac; pmLL = posterior margin of lateral lobes; PP = posterior part of spermathecae. Scale bars: 0.5 mm (equal for **A–C**); 5 mm (equal for **D** and **E**).

**Female** (WYSZY007) (Fig. 1D and E). Total length 18.9. Prosoma 9.2 long, 8.4 wide, anterior width of prosoma 4.5. Opisthosoma 9.7 long, 5.5 wide. Eye sizes and interdistances: AME 0.37, ALE 0.59, PME 0.44, PLE 0.58, AME–AME 0.54, AME–ALE 0.20, PME–PME 0.76, PME–PLE 0.99, AME–PME 0.70, ALE–PLE 0.71, CH AME 0.57, CH ALE 0.61. Spination: Palp: 131, 101, 2121, 1014; Fe: I–III 323, IV 321; Pa: I–IV 101; Ti: I–IV 2026; Mt: I–II 1014, III 2024, IV 3036. Measurements of palp and legs: Palp 11.7 (3.2, 2.0, 2.5, 4.0), I 26.9 (7.4, 3.9, 6.7, 6.8, 2.1), II 29.6 (8.2, 4.3, 7.7, 7.1, 2.3), III 24.2 (7.3, 3.4, 6.0, 5.5, 2.0), IV 25.9 (7.5, 2.7, 6.6, 6.9, 2.2). Leg formula: II-I-IV-III. Cheliceral furrow with three anterior and four posterior teeth and with ~ 55–60 denticles.

**Colouration in ethanol** (Fig. 1D and E). Carapace dark yellowish to brown, with light brown submarginal transversal band at posterior part. Median band of carapace bright yellowish-brown, lateral bands dark brown and not distinctly delimited to median band. Fovea and radial furrows distinctly marked. Chelicerae dark brown. Sternum reddish-brown, margin distinctly lighter. Endites and labium brown, both with distal parts brighter. Legs dark yellowish-brown, covered by dark spots and short spines. Dorsal abdomen anteriorly yellowish-brown, posteriorly dark brown and with three pairs of inconspicuous black dots on each side; ventral abdomen reddish-brown with irregular pattern and two diagonal yellow lines between epigastric furrow and spinnerets.

**Copulatory organ** (Fig. 1A–C). Epigynal field ca. 1.5× wider than long; anterior margin distinct, trilobate, anterolaterally with two small u-shaped incisions separated by ca. 7.5× widths; anterior bands (AB) curved, long and distinct, nearly ‘∫’-shaped, situated at the two incisions. Lobal septum (LS) wide, anterior part about 1/6 width of epigynal plate, gradually wider to the posterior. Lateral lobes (LL) subtriangular, nearly as long as wide, fused along a fovea (LF) on the central axis; lobal fovea (LF) longitudinal, about 2/3 length of lobal septum, clothed with short, dense hairs; anterior margins of lateral lobes (amLL) distinctly procurved and delimited; posterior margins of lateral lobes (pmLL) slightly procurved and bilobed, with small median incision. Internal ducts (ID) thick, nearly 1/6 width of epigynal plate, running parallel along median line, nearly as long as epigyne length. Glandular appendages (GA) thumb-like, slightly inflated, extending obliquely. Posterior part of spermathecae (PP) globular, moderately large, widely separated by about 3× diameters. Fertilisation ducts (FD) acicular, membranous, located on dorsal-basal surface of spermathecae. Membranous sac (MS) between fertilisation ducts, more or less disc-shaped.

**Figure 2.**
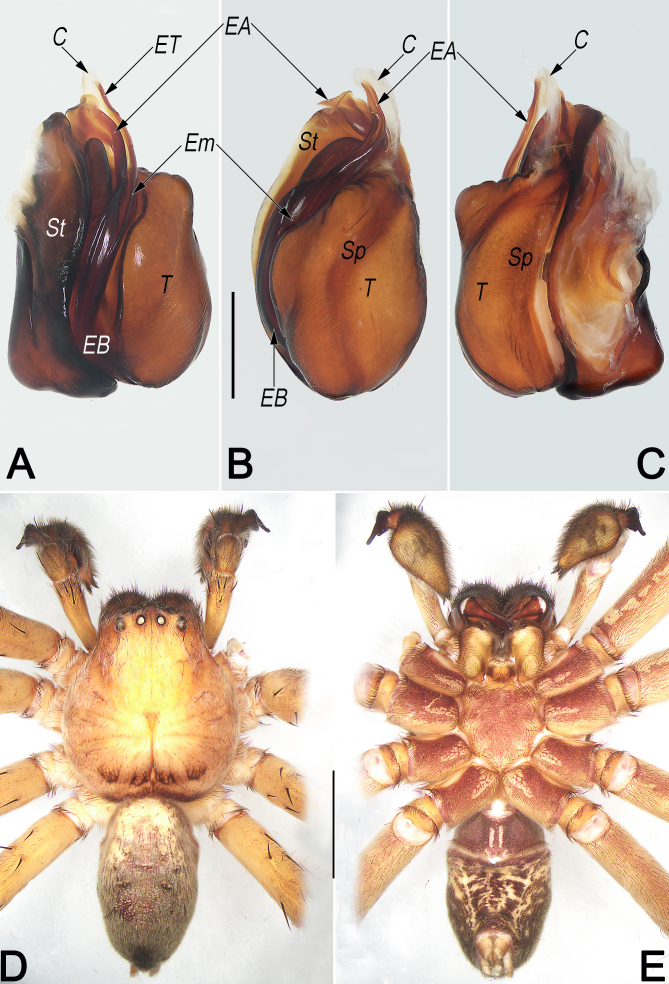
*Sinopodacurva* Zhong, Jäger, Chen & Liu, 2019 from Wuyishan National Nature Reserve. male, palpal bulb (**A–C**) and habitus (**D, E**). **A** prolateral; **B** ventral; **C** retrolateral; **D** dorsal; **E** ventral. Abbreviations: C = conductor; EA = embolic apophysis; EB = embolic base; Em = embolus; ET = embolic tip; Sp = spermophor; St = subtegulum; T = tegulum. Scale bars: 1 mm (equal for **A–C**); 5 mm (equal for **D** and **E**).

**Male** (WYSZY006) (Fig. 2D and E). Total length 17.6. Prosoma 9.0 long, 8.6 wide, anterior width of prosoma 5.4. Opisthosoma 8.6 long, 5.3 wide. Eye sizes and interdistances: AME 0.46, ALE 0.55, PME 0.42, PLE 0.57, AME–AME 0.39, AME–ALE 0.18, PME–PME 0.56, PME–PLE 0.81, AME–PME 0.57, ALE–PLE 0.65, CH AME 0.31, CH ALE 0.33. Spination: Palp: 131, 101, 1021; Fe: I–III 323, IV 321; Pa: I–IV 101; Ti: I 2426, II–IV 2326; Mt: I–III 2024, IV 3036. Measurements of palp and legs: Palp 12.4 (4.0, 1.9, 2.1, 4.4), I 34.6 (9.0, 4.3, 8.7, 9.4, 3.2), II 36.5 (9.6, 4.5, 9.4, 10.0, 3.0), III 27.8 (8.0, 3.9, 7.0, 6.7, 2.2), IV 30.1 (8.6, 2.9, 7.7, 8.2, 2.7). Leg formula: II-I-IV-III. Cheliceral furrow with three anterior and four posterior teeth and with ~ 48–55 denticles.

**Pattern and colouration** (Fig. 2D and E). As in females, but body slightly lighter (see Zhong et al. (2019) for description of others).

**Figure 3.**
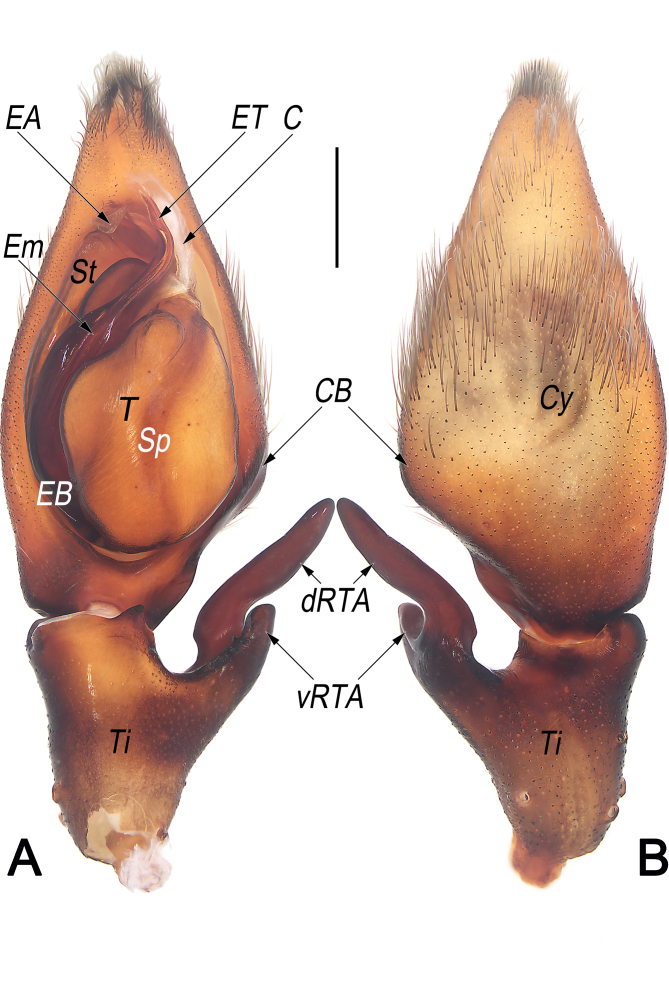
Male palp of *Sinopodacurva* Zhong, Jäger, Chen & Liu, 2019 from Wuyishan National Nature Reserve. **A** ventral; **B** dorsal. Abbreviations: C = conductor; CB = cymbial bulge; Cy = cymbium; dRTA = dorsal branch of RTA; EA = embolic apophysis; EB = embolic base; Em = embolus; ET = embolic tip; Sp = spermophor; St = subtegulum; T = tegulum; Ti = palpal tibia; vRTA = ventral part of RTA. Scale bar: 1 mm (equal for **A** and **B**).

**Figure 4.**
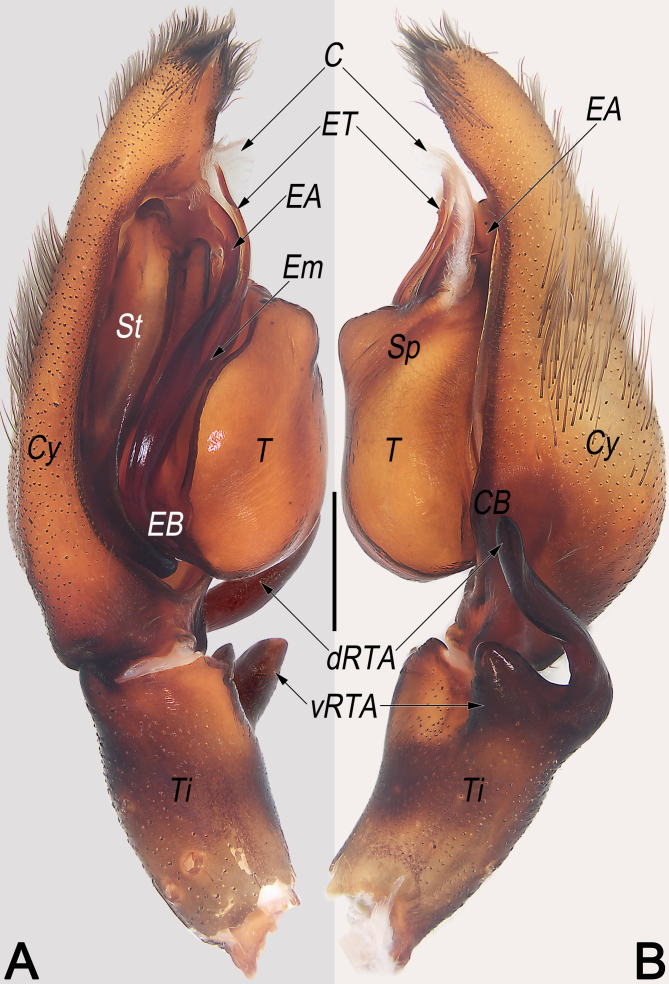
Male palp of *Sinopodacurva* Zhong, Jäger, Chen & Liu, 2019 from Wuyishan National Nature Reserve. **A** prolateral; **B** retrolateral. Abbreviations: C = conductor; CB = cymbial bulge; Cy = cymbium; dRTA = dorsal branch of RTA; EA = embolic apophysis; EB = embolic base; Em = embolus; ET = embolic tip; Sp = spermophor; St = subtegulum; T = tegulum; Ti = palpal tibia; vRTA = ventral part of RTA. Scale bar: 1 mm (equal for **A** and **B**).

**Palp** (Fig. 2A–C, Fig. 3A, B, Fig. 4A and B). Tibia (Ti) moderately long, ca. 1/2 cymbium length, with bifurcated retrolateral apophysis (RTA) arising medially and U-shaped in retrolateral view, both ventral and dorsal branches distinctly protruding: dorsal branch (dRTA) finger-like, slightly curved and tapering, ca. 2/3 of tibia length, extending to cymbial base; ventral branch (vRTA) subtriangular in retrolateral view, relatively short, ca. 1/2 length of dRTA, apex round. Cymbium (Cy) ca. 2× longer than wide, retrolaterally with indistinct bulge (CB). Tegulum (T) oval, ca. 1.4× longer than wide, posteriorly slightly bulged, slightly excavated on prolatero-apical side to accommodate embolus (Em) and conductor (C); spermophor (Sp) indistinct and slightly curved, (-shaped in ventral view). Embolus (E) long, nearly Ƨ-shaped in ventral view, ca. 1.5× longer than tegular length; the embolic base (EB) situated prolatero-proximal on tegulum (ca. 7–8 o’clock on tegulum); free part of the embolus (Em) slender and filamentous; embolic tip (ET) apically slightly widen, arrowhead-shaped, terminated at ca. 12 o’clock position. Embolic apophysis (EA) nearly as long as as embolus, extending alongside embolus, with hyaline apex slightly widen and folded. Conductor (C) membranous, ca. 2/5 of embolus length, sligtly curved, extending obliquely, arising at ca. 1 o’clock position from tegulum, terminating at ca. 12 o’clock position; conductor (C) proximally narrowed, directed prolaterally and apically beyond embolic tip (ET).

##### DNAbarcodes

5'ATGAATAATTTGAGTTTTTGACTTCTTCCTCCTTCTTTAATATTGTTGTTTGTTTCTTCTATAGTTGAAGTGGGAGTGGGAGCGGGGTGGACTATTTATCCTCCTTTGGCTTCTGTGATTGGGCATGCTGGTAGATCTGTGGATTTTGCTATTTTTTCTTTGCATTTAGCTGGAGCTTCTTCTATTATGGGTGCTATTAATTTTATTTCTACTATTATTAATATACGTTCTCCTGGAATAAGAATAGAAAGGGTTCCTTTATTTGTGTGATCTGTATTGATTACTGCGGTTTTATTATTATTGTCTTTACCGGTTTTAGCTGGTGCTATTACTATGCTTTTGACTGATCGAAATTTTAATACTTCTTTTTTTGATCCTGCTGGAGGAGGTGATCCTGTTTTGTTTCAACATTTATTTTGGTTTTTTGGGCATCCTGAGGTTTATATTTTAATTTTACCTGGATTTGGTATTGTGTCTCATGTGATTAGCGGTTCAGTAGGTAAACGGGAGCCATTTGGAAGTTTAGGAATGATTTATGCTATGGTTGGGATTGGGGGAATAGGGTTTGTGGTATGAGCTCATCATATATTTTCTATTGGAATAGATGTGGATACTCGTGCTTATTTTACTGCTGCTACTATAATTATTGCTGTGCCTACTGGAATTAAAATTTTTAGATGAATGGCGACCCTTCATGGATCTTATTTTAAAGTAGATACTTCATTAATGTGAAGAATTGGTTTTGTGTTTTTATTTACTTTAGGTGGAATTACTGGGGTAGTTCTTTCTAATTCTTCTTTAGATATTATTCTTCATGATACTTATTATGTAGTTGCTCATTTTCATTATGTGTTGAGAATAGGTGCTGTGTTTGCTATTATAGCTGGAGTTATTTATTGATTTCCTTTATTTTTTGGGGTGGTTTTGAGAGAAAAGAAAACTAAATTGCAATTTTATGTTATGTTTATTGGAGTTAATATAACTTTTT3'

(WYSZY006; male; Genebank accession number: PV341275).

5'ATGAATAATTTGAGTTTTTGACTTCTTCCTCCTTCTTTAATATTGTTGTTTGTTTCTTCTATAGTTGAAGTGGGAGTGGGAGCGGGGTGGACTATTTATCCTCCTTTGGCTTCTGTGATTGGGCATGCTGGTAGATCTGTGGATTTTGCTATTTTTTCTTTGCATTTAGCTGGAGCTTCTTCTATTATGGGTGCTATTAATTTTATTTCTACTATTATTAATATACGTTCTCCTGGAATAAGAATAGAAAGGGTTCCTTTATTTGTGTGATCTGTATTGATTACTGCGGTTTTATTATTATTGTCTTTACCGGTTTTAGCTGGTGCTATTACTATGCTTTTGACTGATCGAAATTTTAATACTTCTTTTTTTGATCCTGCTGGAGGAGGTGATCCTGTTTTGTTTCAACATTTATTTTGGTTTTTTGGGCATCCTGAGGTTTATATTTTAATTTTACCTGGCTTTGGTATTGTGTCTCATGTGATTAGCGGTTCAGTAGGTAAACGGGAGCCATTTGGAAGTTTAGGAATGATTTATGCTATGGTTGGGATTGGGGGAATAGGGTTTGTGGTATGAGCTCATCATATGTTTTCTATTGGAATAGATGTGGATACTCGTGCTTATTTTACTGCTGCTACTATAATTATTGCTGTGCCTACTGGAATTAAAATTTTTAGATGAATGGCGACCCTTCATGGATCTTATTTTAAAGTAGATACTTCATTAATGTGAAGAATTGGTTTTGTGTTTTTATTTACTTTAGGTGGAATTACTGGAGTAGTTCTTTCTAATTCTTCTTTAGATATTATTCTTCATGATACTTATTATGTAGTTGCTCATTTTCATTATGTGTTGAGAATAGGTGCTGTGTTTGCTATTATAGCTGGAGTTATTTATTGATTTCCTTTATTTTTTGGGGTGGTTTTGAGAGAAAAGAAAACTAAATTGCAATTTTATGTTATGTTTATTGGAGTTAATATAACTTTTT3'

(WYSZY007; female; Genebank accession number: PV341276).

5'ATGAATAATTTGAGTTTTTGACTTCTTCCTCCTTCTTTAATATTGTTGTTTGTTTCTTCTATAGTTGAAGTGGGAGTGGGAGCGGGGTGGACTATTTATCCTCCTTTGGCTTCTGTGATTGGGCATGCTGGTAGATCTGTGGATTTTACTATTTTTTCTTTGCATTTAGCTGGAGCTTCTTCTATTATGGGTGCTATTAATTTTATTTCTACTATTATTAATATACGTTCTCCTGGAATAAGAATAGAAAGGGTTCCTTTATTTGTGTGATCTGTATTGATTACTGCGGTTTTATTATTATTGTCTTTACCGGTTTTAGCTGGTGCTATTACTATGCTTTTGACTGATCGAAATTTTAATACTTCTTTTTTTGATCCTGCTGGAGGAGGTGATCCTGTTTTGTTTCAACATTTATTTTGGTTTTTTGGGCATCCTGAGGTTTATATTTTAATTTTACCTGGCTTTGGTATTGTGTCTCATGTGATTAGCGGTTCAGTAGGTAAACGGGAGCCATTTGGAAGTTTAGGAATGATTTATGCTATGGTTGGGATTGGGGGAATAGGGTTTGTGGTATGAGCTCATCATATATTTTCTATTGGAATAGATGTGGATACTCGTGCTTATTTTACTGCTGCTACTATAATTATTGCTGTGCCTACTGGAATTAAAATTTTTAGATGAATGGCGACCCTTCATGGATCTTATTTTAAAGTAGATACTTCATTAATGTGAAGAATTGGTTTTGTGTTTTTATTTACTTTAGGTGGAATTACTGGGGTAGTTCTTTCTAATTCTTCTTTAGATATTATTCTTCATGATACTTATTATGTAGTTGCTCATTTTCATTATGTGTTGAGAATAGGTGCTGTGTTTGCTATTATAGCTGGAGTTATTTATTGATTTCCTTTATTTTTTGGGGTGGTTTTGAGAGAAAAGAAAACTAAATTGCAATTTTATGTTATGTTTATTGGAGTTAATATAACTTTTT3'

(WYSZY008; female; Genebank accession number: PV341277).

#### Diagnosis

Females of *S.curva* can be easily distinguished from those of all other congeners, with the exception of *Sinopodaexspectata* Jäger & Ono, 2001, by their similar internal ducts (ID), which are distinctly thick, with a diameter larger than that of glandular appendages (GA) and posterior part of spermathecae (PP) (Fig. 1C and Grall and Jäger (2020): 23, fig. 12b) (vs. ID thinner than either GA or PP), but can be distinguished from the latter by: (1) lobal septum (LS) with a longitudinal fovea (LF) on the central axis (vs. fovea absent) (cf. Fig. 1A and B and Grall and Jäger (2020): fig. 12a); (2) posterior margins of lateral lobes (pmLL) bilobed, with small median incision (vs. not bilobed, without median incision) (cf. Fig. 1A and B and Grall and Jäger (2020): fig. 12a); (3) glandular appendages (GA) thumb-like, slightly inflated (vs. fingertip-like, not inflated) (cf. Fig. 1C and Grall and Jäger (2020): fig. 12b). Males of *S.curva* can be distinguished from all other *Sinopoda* species by the combination of the following characters: (1) RTA U-shaped in retrolateral view (Fig. 4B) (vs. not U-shaped); (2) tip of embolic apophysis (EA) hyaline, slightly widened and folded (Fig. 3A, Fig. 4A, Fig. 2A and B) (vs. not widened or folded).

#### Distribution

**Figure 5.**
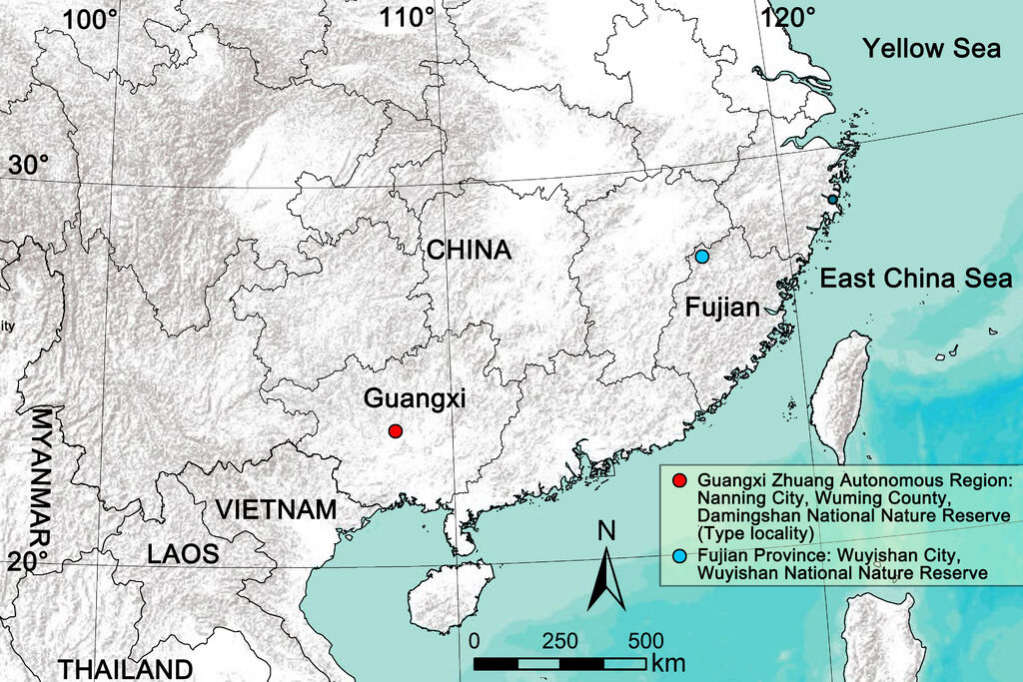
Distribution records of *Sinopodacurva* Zhong, Jäger, Chen & Liu, 2019.

China (Guangxi, Fujian) (Fig. 5). The new collections extend the known range of this species by ~ 1060 km to the northeast (Wuyishan) from the type locality (Damingshan).

#### Biology

The species was found in leaf litter and on tree bark.

## Supplementary Material

XML Treatment for Sinopoda curva
